# Elements of General Pathology: A Practical Treatise on the Causes, Forms, Symptoms, and Results of Disease

**Published:** 1848-05

**Authors:** 


*Elements of General Pathology: a Practical Treatise on the Causes, Forms, Symptoms, and Results of Disease.* By ALFRED STILLE, M. D., lecturer on Pathology and practice of Medicine, etc. Philadelphia: Lindsay and Blakiston, 1848. 8vo. pp. 483.

This work constitutes the second number of the Medical Practitioner's and Student's Library, consisting of a series of original treatises on the several departments of Medical science, of which mention was made in the last number of this Journal.

The author commences the work with an introductory essay on "medical truth—its nature, sources, and means of attainment," presenting some appropriate and valuable remarks on a subject to which more reflection might be profitably given, than *is* always bestowed by medical students and practitioners.

*Part first,* treats of *ætiology,* to which sixty-five pages are allotted; *part second,* of the "*general phenomena, theory and classification of diseases, general diagnosis and prognosis,*" covering ninety seven pages. As a specimen of the style and other characters of the work, we quote the concluding portion of the chapter on the theory of disease. The reader will perceive that Dr. S. adopts an eclectic pathology, avoiding the extremes of humoralism and solidism. In the justness of his views all sound pathologists must concur:—

"In the midst of these various theories of the origin of disease, the rational enquirer must be seriously embarrassed, if called upon to adopt some one of them exclusively; but if he has taken as his guide the rule of incorporating nothing into his scientific creed, but what results from a strict induction of facts, his perplexity need not be prolonged. He will then find that each theory rests upon a certain number of truths, but none upon a sufficient number to give it stability. He must speedily recognize the fact that a large number of the phenomena of life are explicable by chemical laws, and by them only, but that, at the same time, these laws are maintained in operation by the superintendence of vitality, a power which thus not only sustains them, but also on certain occasions subverts them. He must also observe that some functions are performed according to purely mechanical laws, but that their moving power is the principal of life, a force possessed of attributes differing from those of gravity, elasticity, and the other mechanical powers. He must admit that in some diseases, the starting point is the solids, as where they are injured by violence; but he must, at the same time, acknowledge that not a few maladies called general, are directly owing to the introduction of some foreign matter into the blood-vessels, and do therefore originate in the fluids. He cannot deny that even in the first mentioned case, the blood speedily undergoes an alteration which is proportioned to the local hurt, and must, with better reasons, be accepted as the cause of the general symptoms, than the local lesion itself; and that in the second case the original alteration of the fluids is usually followed by a more or less general change of structure in the solids. Finally, he cannot refuse to believe, that some affections commence by an impression upon the mind, the senses, or the general sensibility ; but that amongst all of these, scarcely one remains confined to the system where it originated, but sooner or later implicates the rest. In a word, he beholds in man, a machine indeed, but one composed of various materials, which unlike those of other machines, are perpetually acting and reacting on one another ; not only an aggregate of reacting elements, but one endowed with life, a power to which the ordinary laws of those elements are wholly subordinated, and whose own mode of being and laws are wrapped in profound mystery ; not only a living animal, but a rational one, possessed of a soul, which, with peculiar faculties and susceptibilities, is capable of modifying the usual succession of phenomena resulting from the co-operation or conflict of the chemical, mechanical, and vital laws which govern the merely animal portion of the creature."

*Part third* treats of *semeiology* and embraces more than one-third of the work—192 pages. The following remarks on cyanosis we find marked for quotation:—

"A less intense color, a bluish or as it is called, *cyanotic* hue, is frequently met with in new-born infants and others in a state of asphyxia, when any obstacle to hæmatosis exists in the lungs, causing a stagnation of blood in these organs ; in severe attacks of cholera ; and, above all, in the disease whose principal symptoms is blueness of the skin, and which is hence called cyanosis. In all of these examples, the cutaneous discoloration is due entirely to congestion of the venous capillaries, in consequence of some obstacle to the return of the blood to the heart. This explanation of the phenomena of cyanosis is not that which has generally been accepted. It has for a long time been taught that in all cases of congenital cyanosis, the foramen ovale of the heart remains open, and permits the dark venous blood of the right side of the heart to mingle with the bright arterial blood of the left side. This admixture it has been alleged, forms a dark colord fluid which communicates to the skin a cyanotic hue. Louis was the first person of recent times to call this doctrine in question ; and as early as 1826, he showed that a communication between the two sides of the heart might exist without the concurrence of the cyanosis, and that on the other hand this color might arise without any malformation or disease of the heart whatever. He further stated the conclusion that many cases of imperfection were owing to congestion, produced by an obstacle to the venous circulaton. These views were more or less fully adopted by several distinguished medical men in France; but the doctrine previously taught continued to prevail. In 1843 Dr. Moreton Stille, in his inaugural thesis, proved by the analysis of a large series of cases, that the cause of cyanosis was *uniformly* either contraction, obstruction, or imperforation of the pulmonary artery, or else some physical impediment to the natural course of the blood possessing a similar mode of action, such as concentric hypertrophy of the right ventricle, singleness of the heart with contraction of the auriculo-ventricular opening, &c. This conclusion has since received ample confirmation, and especially from the investigation of Dr. Norman Cheevers of London.

It is remarkable that the explanation given by Morgagni of a case of cyanosis witnessed by him, and which tallies precisely with the results of the laborious analysis above alluded to, and should have, for so long a time, failed to convince pathologists of their error in attributing to admixture of arterial and venous blood the phenomena of cyanosis. Morgagni, after describing the case, and the dissection which he made upon the patient's death, disclosing a contracted and ossified pulmonary artery, declares this lesion to have been the cause of the symptoms; for, he remarks, "the contraction of the pulmonary artery allowed a very diminished quantity of blood to pass through it and the corresponding vein to the left side of the heart, so that less blood than natural was circulated through the body; and on the other hand, it caused the blood to accumulate and stagnate in the right ventricle, then in the right auricle, and finally in all the veins, *whence resulted the discoloration of the skin*, the dilation of the right cavities, and the opening of the foramen ovale whose valve was pressed by more blood upon the right than upon the left side." The confirmation of this ingenious statement by the result of an analysis of all the cases of cyanosis that could be collected, is a beautiful illustration of the uniformity of nature's laws, and a proof of the rare sagacity of the founder of the pathological school."

With reference to the *impulse of the heart*, Dr. Stille dissents from the generally received opinion that it is due to the shock of the apex against the parietes of the thorax during the systole of the ventricles. He attributes it to the diastole of the ventricles, which he appears to think is attributable not to an active expansibility inherent in the ventricles, but to the auricular contractions, forcing the blood into the ventricular cavities. He considers the force of the impulse and diastole to be proportioned to the power with which the auricle expels its contents. In proof of this view, he adduces the fact that an increased impulse does not always coexist with ventricular hypertrophy, but, on the other hand, in some cases of that affection, the impulse is diminished. He avers that analyses of cases, respectively, of hypertrophy in which the impulse is increased, and of cases in which it is diminished, will show that in the former the *auricles* are hypertrophied, while in the latter they are unaffected. He does not, however, give such analyses, nor state that they have even been made. The reasons for rejecting the opinion commonly entertained on the subject, seem to us far from conclusive. A well known fact to which he makes no reference, suffices to disprove his hypothesis:—It is a matter of common observation, that the heart, especially of cold-blooded animals, continues to pulsate for a certain period after being removed from the body. What causes the ventricular diastole under these circumstances? This fact certainly establishes the power of the ventricles to dilate irrespective of distension of the blood received from the auricles, for the auricles, in this case, are empty. The concurrence of hypertrophy and diminished impulse does not involve the necessity of such an hypothesis. Diminished quantity, and altered quality of the blood, and other circumstances, in some instances, may prevent an increased power of contraction of the ventricle, proportioned to its increase in bulk:

In presenting a summary of the physical signs of valvular lesions, the author omits to point out with particularity the means of determining the location of the lesions—whether in the arterial or auriculo-ventricular orifices. He speaks incidentally of the evidence to be derived from the points of location at which the maximum intensity of the bellows murmur is heard, but he makes no mention of the differences in pitch or key which the murmur presents in the two localities. It is true that to settle the location of the lesions is generally not of great practical moment, yet, as a matter of scientific interest, exemplifying the precision of diagnosis, it is deserving attention.

*Part fourth*, concludes the work, and is devoted to *general morbid anatomy*—70 pages. In treating of Entozoa, the author advocates the doctrince of spontaneous, or equivocal generation. In this he is not sustained by prevailing opinion, but we fully concur with him that the evidence against the reception of ova from without the body preponderates greatly over the arguments from analogy in defence of the doctrine *omne vivum ex ovo*. We are aware that it is contended for the existence of prototypes of the intestinal entozoa out of the body. In the reported lectures of Prof. Agassiz, delivered at N. York, that distinguished naturalist declared this fact to have been demonstratively established. But, admitting this to be the case, may we not ask why are they not more abundant and oftener observed out of the body, than within, or proceeding from it? Should they not be *more* abundant without than within the body, if, in the latter instance they are produced from ova, furnished by the former? Is it not more reasonable, under existing circumstances, to attribute their production out of the body, *in the rare instances in which they are said to have been observed*, to ova derived from intestinal worms, than vice versa?

The consideration involved in these questions is submitted in addition to the arguments succinctly stated by Dr. S., which, in the present state of our knowledge, appear to us to be unanswerable.

In concluding this brief notice of Dr. Stille's work, we take pleasure in saying that, in our judgment, it is a creditable performance.

It bears internal evidence that the author is an accurate observer, a careful, as well as diligent student, and a philosophical thinker. It contains much valuable information; it inculcates a truly scientific spirit, and is written in a perspicuous, chaste, and even elegant style. Nevertheless, the work is unsatisfactory from its condensation. Its range of subjects is altogether too extensive for its prescribed limits. The author has made the most of the space allotted to him, but the size of the work is inadequate even for an elementary treatise on the several departments of general patholoy. Should the success of this production be commensurate with its merits, we trust that the author will be encouraged to undertake a more extended and elaborate work, which will afford him an opportunity of doing better justice to himself, as well as to the subjects of which he treats.

